# Outcome harvesting: evaluation of a mentoring program supporting Latino agricultural professionals in the United States

**DOI:** 10.3389/fsoc.2026.1726136

**Published:** 2026-05-11

**Authors:** Renzo Ceme, Pablo Lamino

**Affiliations:** 1 Department of Agricultural Education and Communication, University of Florida, Gainesville, FL, United States; 2 University of Florida Institute of Food and Agricultural Sciences, Gainesville, FL, United States; 3 University of Florida, Gainesville, FL, United States

**Keywords:** AGEAP alumni, cross-cultural support, mentoring programs, outcome harvesting, professional development

## Abstract

**Introduction:**

Mentoring programs play a vital role in supporting international students' academic and professional transitions. The AGEAP USA Mentoring Program was launched in 2020 to assist a Central American university alumni in the United States by fostering professional relationships, offering guidance for graduate studies and job applications, and promoting leadership development. Given the unique challenges faced by international alumni, this study evaluates the program's effectiveness using a culturally responsive lens.

**Methods:**

This qualitative evaluation employed the Outcome Harvesting methodology, which retrospectively identifies changes resulting from interventions. Data were collected through document analysis and semi-structured interviews with 30 participants, including mentors, mentees, and organizers, spanning cohorts from 2020 to 2024. Thematic analysis and triangulation were used to interpret outcomes, supported by memoing and validation meetings with program coordinators.

**Results:**

Findings revealed that mentees benefited from professional development, shared experiences, and expanded professional networks. Mentors reported growth in communication, leadership, and empathy, while organizers developed organizational skills and strengthened alumni connections. Challenges included inconsistent mentee engagement, difficulties in matching mentors and mentees, and logistical barriers. Motivations for participation centered on giving back to the community, improving transition experiences, and reinforcing a sense of belonging.

**Discussion:**

The program demonstrated strong potential to support international alumni, though structural improvements are needed to enhance engagement and sustainability. Recommendations include refining matching processes, clarifying expectations, and implementing centralized tools for coordination and training.

## 1 Introduction

### 1.1 Defining mentoring and core components

Mentoring is an intentional process that fosters a developmental relationship between two individuals, typically a more experienced mentor and a less experienced mentee (Al-Aqeel and Alhumaid, 2024; Nuis et al., 2023). Through this relationship, mentors provide various forms of support, including career guidance, emotional encouragement, psychosocial assistance, and academic help, all aimed at promoting the mentee's personal growth, skill development, and professional advancement (Al-Aqeel and Alhumaid, 2024; Nuis et al., 2023).

An effective mentoring program is built upon several core components that contribute to its success. These include goal setting, open communication, constructive feedback and reflection, advocacy, and role modeling (Hairon et al., 2020; Hattie and Timperley, 2007; Hong and Matsko, 2019; McHugh and Gruppen, 2021; Moloney et al., 2023; Salim, 2021; Speizer, 1981; Welsh and Dixon, 2016).

To begin with, establishing clear objectives is essential for guiding the mentoring relationship and aligning expectations (Hairon et al., 2020; Salim, 2021). Open communication plays a critical role in fostering trust and mutual understanding between mentors and mentees (Hong and Matsko, 2019; McHugh and Gruppen, 2021). Constructive feedback and opportunities for reflection enable mentees to recognize their progress and identify areas for growth (Hattie and Timperley, 2007; Moloney et al., 2023).

Mentors often serve as advocates, helping mentees navigate institutional systems and gain access to valuable opportunities (Salim, 2021; Welsh and Dixon, 2016). Moreover, effective mentors act as role models, demonstrating professional behavior, ethical standards, and sound decision-making practices that mentees can emulate (Salim, 2021; Speizer, 1981).

### 1.2 Mentoring international students: challenges and needs

Mentoring individuals from other countries requires careful consideration of various factors that influence their academic and personal success (Adebayo and Sunderman, 2023; Kirshteyn and Uysal, 2022; White et al., 2024). These factors include cultural adjustment, language barriers, emotional and social support, and the ability to navigate unfamiliar or complex institutional systems (Patel et al., 2024; Wilczewski and Alon, 2023). With approximately 1.1 million international students enrolled in U.S. higher education institutions in 2024 (Martel and Kokodyniak, 2025), mentoring programs must evolve to meet the increasing needs of this population.

To promote a more equitable and enriching educational experience, mentoring should go beyond traditional academic guidance (White et al., 2024). It must adopt holistic, culturally responsive approaches that actively support students in overcoming communication challenges, building social connections, and accessing institutional resources (Adebayo and Sunderman, 2023; Kirshteyn and Uysal, 2022; Patel et al., 2024; White et al., 2024; Wilczewski and Alon, 2023).

### 1.3 The Central American University context

International students bring with them rich academic backgrounds and cultural experiences shaped by educational systems around the world (White et al., 2024). A notable example is the Central American University in Honduras, an agricultural institution with over 10,000 graduates, Central American University has become a leader in preparing students for global academic and professional mobility through rigorous programs in agricultural science, agribusiness management, food science and technology, and environmental science and development (Universidad Zamorano, 2025a; Wiedenmann, 2024).

Currently, Central American University also offers specialized master's degree programs in agribusiness, coffee growing and business, and nutrition and community development, further enhancing its role in shaping globally competent professionals (Universidad Zamorano, 2025a). This University hosts undergraduate and graduate students from about 19 countries (Universidad Zamorano, 2025b). After graduating from Central American University, it is common for alumni to pursue international professional experiences, and the U.S. is a popular destination for that. In addition, Central American University alumni associations are based in countries; they are called AGEAP.

### 1.4 Overview of the AGEAP USA mentoring program

The presence of alumni from institutions like Central American University in U.S. educational settings highlights the critical need for culturally responsive mentoring. These students often encounter distinct challenges, including cultural adjustment, language barriers, and navigating unfamiliar academic systems (Adebayo and Sunderman, 2023; Kirshteyn and Uysal, 2022; White et al., 2024). Recognizing these needs, a group of Central American University alumni within AGEAP USA launched the Mentoring Program in 2020 to support the professional and personal development of fellow Central American University graduates. The initiative aims to foster meaningful connections among alumni and provide guidance through shared experiences and insights from seasoned mentors, creating a supportive network that bridges cultural and institutional gaps.

### 1.5 Sociological framing

The AGEAP USA Mentoring Program is situated within sociological perspectives that view mentoring and work as embedded in relational and participatory social structures. Contemporary scholarship highlights a shift from traditional, hierarchical senior–junior models toward developmental networks, peer-to-peer, and cross-cultural mentoring that emphasize shared learning and reduced power distance in increasingly diverse organizational contexts (Emerson, 1962; Montgomery and Page, 2025). Guided by this orientation, our conceptual framework draws on two complementary models: relational mentoring, which theorizes mentoring as a mutually developmental, trust-based relationship (Emerson, 1962; Ragins, 2012, 2016); and communities of practice (CoP), which not only conceptualizes learning as identity and competence development through participation in shared practices but also provides an explanation how engagement in collective activities can generate progressive shifts in participation, confidence, and professional identity (Ataizi, 2012; Lave and Wenger, 1991). Together, these models clarify the mechanisms by which program activities may foster developmental change in an alumni-to-alumni structure.

#### 1.5.1 Relational mentoring

This program aligns with relational mentoring theory, which conceptualizes mentoring as an interdependent and mutually developmental relationship characterized by trust, reciprocity, and shared growth (Emerson, 1962; Ragins, 2012, 2016). Applied to the AGEAP USA mentoring program, this perspective provides a lens for understanding how structured, relationship-centered interactions may cultivate psychological safety, mutual learning, and skill development while reducing power distance relative to hierarchical models (Ragins, 2016; Van Der Kloot et al., 2025). Through ongoing, reciprocal engagement, participants can co-construct knowledge, exchange social support, and strengthen professional identity, positioning relational processes as a plausible mechanism for developmental change within the program's alumni-to-alumni structure.

#### 1.5.2 Community of practice

Communities of Practice (CoP) theory describes learning as a social process where individuals develop expertise and identity through participation in shared activities (Ataizi, 2012; Lave and Wenger, 1991). In the CoP model, moving from “legitimate peripheral participation” to more central roles involves access to knowledgeable peers, informal norms, and authentic practice (Lave and Wenger, 1991). In the AGEAP USA mentoring program, this developmental journey connects activities to outcomes (Bizzi, 2015; Zamorano Colombia, 2021). Structured mentor–mentee meetings, peer reflection, and collaborative problem-solving help participants access tacit knowledge, interpret norms, and boost confidence in navigating academic and professional settings (Adkisson et al., 2020). These lead to short-term gains like clearer career paths, increased confidence, and social support. Over time, these improvements result in greater professional agency, expanded networks, and better navigation of institutional systems. Ultimately, participants achieve long-term benefits, including career advancement, integration into professional communities, and stronger alumni ties (Holland, 2018).

### 1.6 Purpose of the study and research questions

Since the program was introduced in 2020, no formal evaluation has been conducted. Thus, it is essential to assess its impact through a proper evaluation. For this reason, the purpose of this study is to understand the effectiveness and impact of the AGEAP USA mentoring program on the professional and personal growth of its participants, and to identify the benefits and challenges perceived by mentors, mentees, and program organizers. The following questions guide this study:

1. How do participants perceive benefits from the AGEAP USA Mentoring Program?
2. What challenges did participants encounter during their involvement in the AGEAP USA Mentoring Program?
3. What motivated Central American University alumni to participate in the AGEAP USA Mentoring Program from 2020 to 2024?
4. To what extent did the AGEAP USA Mentoring Program achieve its stated objectives from 2020 to 2024?

## 2 Methods

### 2.1 Program description

The AGEAP USA Mentoring Program has supported about 370 participants over its first 5 years (2020–2024), averaging 70 participants per year, including mentors, mentees, and volunteers. Each cycle engages a volunteer team of 6–10 organizers, who coordinate recruitment, matching, orientation, content development, and evaluation. The program operates annually from January to August and typically enrolls graduate students, early-career professionals, and senior professionals as both mentees and mentors. Since its beginning, the AGEAP USA Mentoring Program's objectives has been: (1) to increase the number of professional relationships among Central American University alumni residing in the United States; (2) to support alumni interested in pursuing graduate education with the U.S. application process; (3) to assist alumni studying in the United States with the transition into the job market; and (4) to provide opportunities for alumni to share their experiences and strengthen their mentoring and leadership skills.

To enroll, mentors and mentees complete a detailed registration form documenting their field of study, professional goals, sector interests, preferred language, and expectations for the mentoring relationship. This information is used by the matching team (typically three to four volunteers) to create pairs based on professional compatibility, shared goals, and relevant expertise. Because the program specifically serves alumni from a shared institutional and cultural background, many pairings naturally reflect common linguistic and cultural experiences; however, cultural identity is not used as a strict matching criterion. Instead, the program emphasizes cultural responsiveness by ensuring that mentors have appropriate contextual knowledge to support mentees as they navigate U.S. academic and professional environments.

The program includes at least a monthly one-on-one meeting (minimum 45 min), an orientation session, and a set of digital workshops covering topics such as résumé development, networking, interviewing, negotiating offers, and adapting to U.S. workplace expectations. Periodic check-ins, communication support, and an end-of-cycle evaluation supplement these activities.

### 2.2 Logic model

To systematically examine how the AGEAP USA Mentoring Program translates its resources and activities into meaningful outcomes, the evaluation is guided by a logic model. Logic models are widely used in program evaluation to articulate a program's underlying theory of change by describing the logical relationships among inputs, activities, outputs, and short-, intermediate-, and long-term outcomes (Centers for Disease Control and Prevention, 2024a,b; W. K. Kellogg Foundation, 2004). By explicitly mapping these connections, logic models provide a transparent representation of how a program is expected to operate and achieve its objectives (Centers for Disease Control and Prevention, 2024a,b).

In addition, logic models help clarify the assumptions, contextual conditions, and external factors that may influence program implementation and effectiveness, thereby strengthening evaluation design and interpretation (Public Health Ontario, 2016; W. K. Kellogg Foundation, 2004). As such, the logic model serves as both an analytic framework and a practical tool for aligning program activities with intended outcomes and guiding subsequent evaluation efforts.

**Figure 1:**
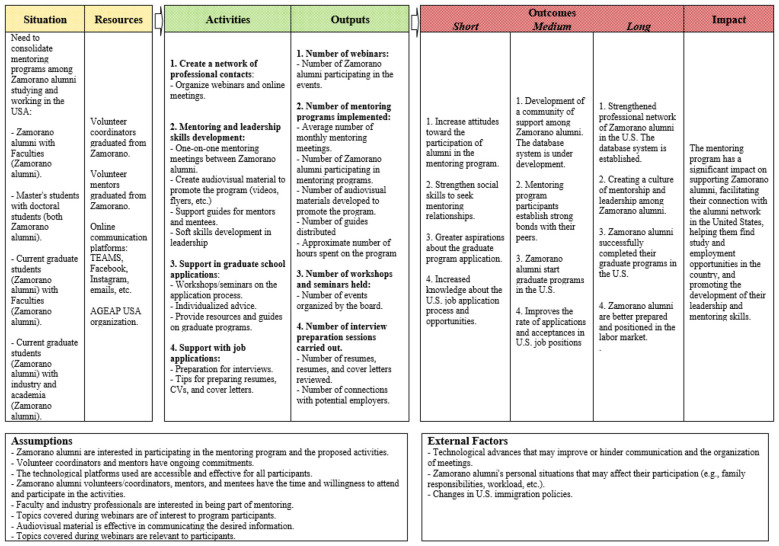
AGEAP USA Mentoring program logic model.

Figure 1 presents the program's Logic Model, developed collaboratively by the authors and the leadership team of the AGEAP USA Mentoring Program. Its creation involved a series of working sessions with program coordinators to clarify and document the program's core resources, activities, outputs, and intended outcomes.

### 2.3 Program evaluation design

Outcome Harvesting is a retrospective evaluation methodology designed to identify and collect evidence of changes, referred to as *outcomes*, that have occurred in contexts where interventions have taken place (Wilson-Grau and Britt, 2012). Unlike approaches that measure progress against predefined indicators, Outcome Harvesting works backward from observed changes to determine whether, and how, an intervention contributed to those changes (Beardmore et al., 2023). Informants who were directly involved in the intervention play a critical role in refining and validating both the outcomes and the narratives of how change occurred.

The methodology involves six iterative phases: (1) design the harvest, (2) collect data and draft outcome descriptions, (3) engage informants, (4) triangulate information, (5) analyze and interpret findings, and (6) support the use of findings. In this study, these phases were adapted to the implementation context of the AGEAP USA mentoring program, which builds on evidence that mentoring interventions can positively influence academic and career outcomes (Crisp and Cruz, 2009). Adaptations were guided by existing literature and documented applications of Outcome Harvesting (Abboud and Claussen, 2016; Carbone et al., 2025).

**Figure 2:**
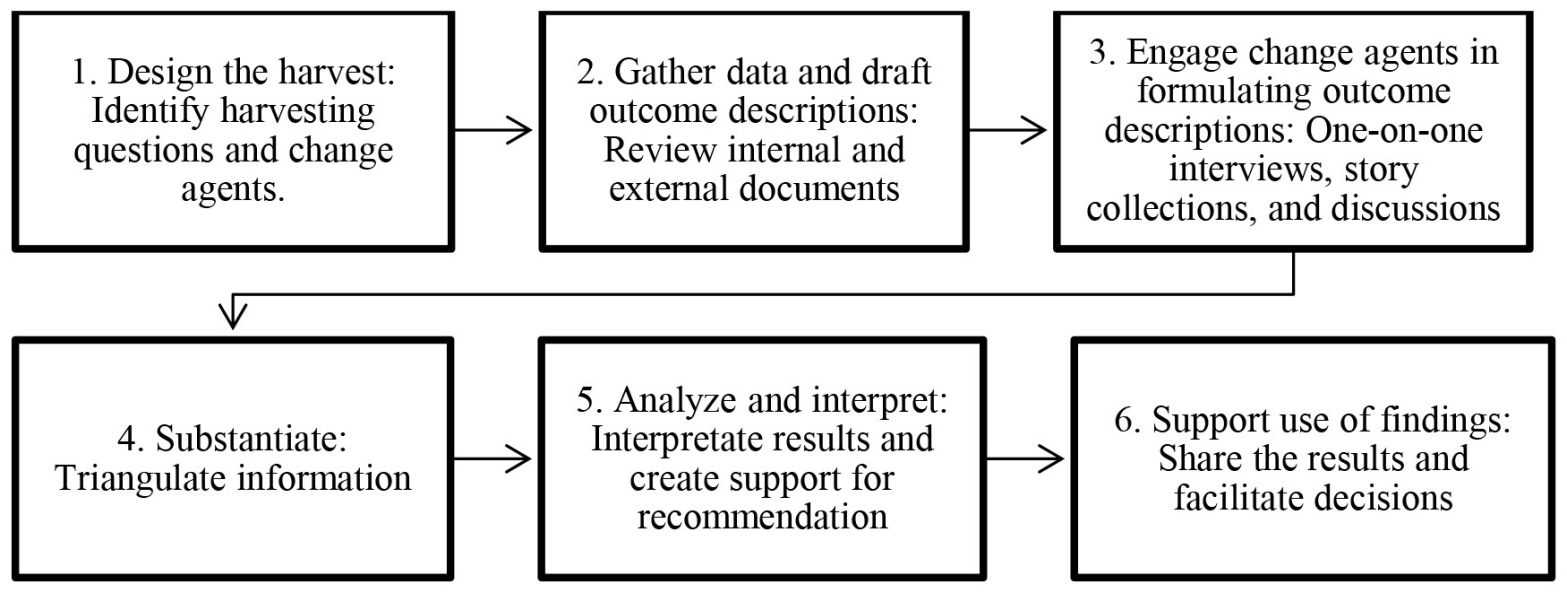
Outcome harvesting process flowchart illustrating the six main key steps of the methodology.

The reporting of phases is integrated throughout the manuscript. Phase 1 (designing the harvest) is described at the end of the introduction and reiterated in the participant recruitment section. Phases 2 and 3 (data collection and informant engagement) are outlined in the data collection section (Wilson-Grau and Britt, 2012). Phase 4 (triangulation of information) is explained in the data analysis section. Phase 5 (analysis and interpretation), which appears in the findings section, and Phase 6 (supporting the use of findings) are presented by the outcome validation. This sequence reflects both the methodology's iterative nature and the practical flow of the study. Figure 2, adapted from Wilson-Grau and Britt (2012), provides a visual summary of the six-phase Outcome Harvesting process as applied here.

### 2.4 Evaluation rationale

Outcome Harvesting was well-suited for this evaluation because the AGEAP USA Mentoring Program did not employ predefined indicators, baseline measures, or a systematic monitoring system across cohorts. These conditions align with the contexts in which Outcome Harvesting is recommended, particularly when traditional indicator-based approaches are not feasible (Wilson-Grau and Britt, 2012).

This study employed Outcome Harvesting as the primary evaluation methodology to examine the intended and emergent outcomes of the AGEAP USA Mentoring Program from 2020 to 2024. Although the program outlined four formal objectives, it lacked predefined indicators and consistent monitoring mechanisms, which are characteristic of the evaluation approach used. Program activities varied across years based on volunteer availability, making the pathways non-linear, inconsistently implemented, and only partially predetermined. These conditions align with contexts for which Outcome Harvesting is recommended, particularly when: outcomes may include unplanned or emergent changes, contribution pathways have not been systematically tracked, and the evaluation aims to understand what changes occurred, rather than solely whether predefined objectives were achieved (Nassar, 2026; United Nations Development Programme, 2026). The ability to capture emergent and unintended outcomes reflects a core strength of Outcome Harvesting, which is specifically designed to identify non-linear, participant-generated changes (Beardmore et al., 2023; Nassar, 2026).

To assess progress toward stated goals, the evaluation sought to capture broader developmental, relational, and community-oriented changes reported by participants. Outcome Harvesting was therefore appropriate because it allows evaluators to work backward from observed change to understand program contributions, identifying intended and unintended outcomes, particularly relational and identity-based changes that the program had not originally articulated, also accommodating expected and emergent forms of learning, growth, and behavior change (Beardmore et al., 2023; Wilson-Grau and Britt, 2012). In addition, this approach allowed us to understand how the program contributed to participants' development, consistent with Outcome Harvesting's emphasis on retrospective, participant-driven outcome identification.

Annual variation in program implementation due to volunteer turnover and shifting coordination structures further contributed to unpredictable pathways of change, reinforcing the need for a retrospective methodology capable of mapping program contributions rather than relying on predetermined causal chains (Abboud and Claussen, 2016). Because the central point of this evaluation is to know the changes that occurred and how participants attributed these changes to the program, Outcome Harvesting provided an appropriate framework for collecting, substantiating, and interpreting participant-validated evidence of both expected and unexpected shifts in behavior, knowledge, relationships, and identity (United Nations Development Programme, 2026).

### 2.5 Participant recruitment

In Phase 1, defining the evaluation purpose, this study aimed to assess the effectiveness and impact of the AGEAP USA Mentoring Program on participants' professional and personal growth by examining the changes resulting from the program and how participants attribute these changes to it. To identify potential participants as key informants, the Principal Investigator [PI] requested access to the program's internal database, which included all individuals who had participated as mentees, mentors, or coordinators across program cohorts from 2020 through 2024.

Participants were recruited from the program's existing database after Institutional Review Board approval IRB ET00045153, which was granted exemption status. Recruitment was carried out via email, with the initial invitation sent by the program's organizational leader. The invitation outlined the study purpose, the significance of contributions, and confidentiality protections. A tailored design approach was used (Steultjens et al., 2023), and two follow-up reminders were sent two weeks after the initial outreach. The goal was to ensure representation across alumni generations, disciplinary backgrounds, and participation roles, which is central to Outcome Harvesting's emphasis on capturing a comprehensive set of outcomes and validating them across informants (United Nations Development Programme, 2026; Wilson-Grau and Britt, 2012). Including mentors, mentees, and organizers across multiple years strengthened triangulation and enhanced the credibility of the harvested outcomes.

### 2.6 Data collection

For Phase 2, gather data and draft outcome descriptions, reviewing internal and external documents. The lead researcher requested and received access to electronic documents related to the mentoring program. These materials included meeting records, mentoring training educational material, and annual and special reports from the mentoring committee to AGEAP USA mentoring.

The exploration of this material supported the rationale for connecting all groups involved in the mentoring program, including mentors, mentees, and coordinators. In Phase 3, change agents were engaged in formulating outcome descriptions through semi-structured interviews. Three interview protocols were developed by the PI in Spanish, tailored to mentors, mentees, and coordinators. These instruments aimed to capture both tangible and intangible outcomes of the program, including personal growth, professional development, and community engagement. To ensure relevance and validity, the instruments were reviewed and refined in collaboration with organizational leaders and a panel of subject-matter experts.

Semi-structured interviews were used because Outcome Harvesting requires participants to describe concrete changes and explain the plausible contribution of the program, which is best accessed through rich, narrative accounts (Blundo Canto, 2026; Wilson-Grau and Britt, 2012). This method also emphasizes collecting outcome information through personal interviews and narrative descriptions to surface both intended and unintended changes and to work backward from lived experiences to contribution analysis (Blundo Canto, 2026; Wilson-Grau and Britt, 2012).

One-on-one interviews were conducted online via Zoom. With participants' informed consent, interviews were recorded and later transcribed. Interviews lasted 30–60 min, depending on the depth of responses and participants' level of involvement. All were conducted in Spanish to reflect participants' language preferences.

Researchers aimed for a sample that would represent variation across participants who held different roles within the AGEAP USA Mentoring Program, including mentors, mentees, and organizers, and across program years. Outcome Harvesting explicitly requires evaluators to gather outcome evidence from multiple stakeholders and to substantiate these outcomes through validation by independent or knowledgeable informants, an approach that mirrors methodological triangulation (Blundo Canto, 2026; United Nations Development Programme, 2026; Wilson-Grau and Britt, 2012). The final sample consisted of 30 participants, aligning with qualitative standards for data saturation, indicating that interviews typically reach saturation when no new information emerges (Guest et al., 2006; Hennink et al., 2017).

Ten unique role combinations were represented, reflecting the initiative's multifaceted nature. Participants' graduation years ranged from 1962 to 2023, spanning a wide generational range and demonstrating the program's reach across alumni networks. While most participants (18 out of 30) were involved in a single cohort year, 12 participants engaged across multiple years of the program, with some contributing consistently from 2020 through 2024. This variation in participation highlights both short-term and sustained engagement with the mentoring program. Table 1 provides a detailed summary of participants' information.

**Table 1:** Participant roles, graduation years, and mentorship participation in the mentoring program.

Participant ID	Mentorship role(s)	Class	Participation year
P1	Mentor	1985	2020–2023
P2	Mentee	2014	2024
P3	Mentee and Mentor	2020	2023–2024
P4	Mentor	1962	2023
P5	Mentor and Organizer	2014	2020
P6	Mentee	2019	2024
P7	Mentor, Mentee, and Organizer	2019	2021–2023
P8	Mentee	2023	2023
P9	Mentor, Mentee, and Organizer	2014	2020–2024
P10	Mentee	2015	2024
P11	Mentor and Organizer	2014	2022–2024
P12	Mentor, Mentee, and Organizer	2014	2020–2021
P13	Mentor	2017	2023
P14	Mentee	2021	2021
P15	Mentee	2023	2024
P16	Mentee and Mentor	2021	2022
P17	Mentor	2014	2024
P18	Mentor and Organizer	2017	2020–2022
P19	Mentee	2021	2021
P20	Mentor and Organizer	2019	2022–2023
P21	Mentor	1998	2024
P22	Mentee	2023	2023–2024
P23	Mentor	1998	2020
P24	Mentee	2023	2023
P25	Mentor	2005	2023
P26	Mentee and Mentor	2022	2023
P 27	Mentor	2014	2023
P28	Mentor	2017	2022
P29	Mentee	2019	2020
P30	Mentee	2022	2022

The findings from the participants' interviews were further supported by the 2024 AGEAP USA internal census, which aimed to improve member engagement and understand participation trends across its initiatives. One survey question asked respondents whether they had participated in the AGEAP USA Mentoring Program. Among the total respondents (*n* = 291), 39 indicated they had participated, 121 reported they had not, and 129 preferred not to answer. For those who had not participated, the survey requested explanations for their decisions, which were used to reinforce the evaluation's findings.

### 2.7 Data analysis

In Phase 4, triangulation occurred across interviews, memos, internal documents, and survey data from the organization's 2024 internal census. Interview transcripts were reviewed multiple times, accompanied by analytical memoing. An iterative coding approach was employed, starting with open coding to extract significant data segments. These initial codes were then systematically grouped into categories reflecting perspectives from mentors, mentees, and organizers. This approach facilitated the development of relevant categories, subcategories, and analytical dimensions (Saldaña, 2016). Subsequently, pattern coding was applied to cluster related codes into overarching themes, enabling a deeper understanding of the interconnections within the data (Morse and Richards, 2002). To enhance reliability, two researchers independently coded the data, resolving any inconsistencies through collaborative discussions held across three consensus meetings.

In addition to the interview data, the lead researcher employed a qualitative technique known as *memoing* (Saldaña, 2016). Memos were written immediately after each interview and during key moments throughout the research process. These memos captured questions, observations, reflections, and emotional responses, offering insights that extended beyond the audio recordings. Memoing enriched the dataset by documenting subjective impressions and situational nuances that might otherwise have been overlooked.

To support the triangulation of the interviews and memos, we linked the data to the document from phase 2 of the internal and external document review. These documents were instrumental in understanding the broader leadership and cultural initiatives connected to the mentoring program. Document analysis was conducted following established qualitative procedures (Bowen, 2009). For documents, coding examined both manifest and latent messages to assess alignment with participant experiences. Saldaña and Omasta (2021) described manifest as readily observable information and latent content as the hidden material that is interpreted. This form of analysis allows for a better understanding of the symbolic meaning of these documents and materials. Materials were compared with interview findings to identify convergence or gaps between institutional structures and lived experiences.

#### 2.7.1 Outcome validation

In Phase 6, validation meetings were held with current program coordinators by sharing draft findings for validation, clarification, and interpretation. The goal was to refine draft outcomes using insights from those directly involved in the intervention. Coordinators reviewed and adjusted outcome statements based on their first-hand experience, and their feedback was incorporated into revised drafts. The information provided by the coordinators ensured alignment across data sources, informed necessary adjustments, and allowed for cross-role and cross-cohort comparisons.

## 3 Results

Through this analytical process, data from mentees, mentors, and program organizers were examined to uncover the perceived benefits of participation across multiple domains, including personal growth, professional development, skill acquisition, and network expansion. The systematic coding and interpretation yielded overarching themes, supported by subthemes that illustrate specific dimensions of participants' experiences. The reporting of the findings aligns with Phase 5 of the Outcome Harvesting methodology, in which the data were systematically analyzed to identify significant outcomes, patterns, and insights.

Although themes and subthemes are presented as analytically distinct categories to enhance clarity, participants' experiences were often interconnected and mutually reinforcing. For example, professional development frequently occurred through networking opportunities, while learning through shared experiences was closely tied to both emotional support and skill acquisition. Thus, the boundaries between subthemes should be understood as fluid rather than discrete, reflecting the holistic and relational nature of mentoring experiences.

The structure and organization of the following themes directly reflect the backward-mapping logic of Outcome Harvesting. Because Outcome Harvesting centers on identifying concrete, participant-described changes, the themes reported here correspond to outcome statements generated during Phases 2–4 of the methodology—data collection, informant engagement, and triangulation (Wilson-Grau and Britt, 2012). These outcomes were further refined through validation meetings with the program coordinator, as per Phase 6, ensuring that the findings reflect both participants' experiences and independently substantiated accounts of change (United Nations Development Programme, 2026). The emergent themes, therefore, reflect clusters of observed changes in behavior, skills, identity, and relationships rather than predetermined evaluative categories, aligning with Outcome Harvesting's emphasis on capturing unexpected, relational, and context-specific outcomes (see Supplementary material) (Beardmore et al., 2023). This methodological alignment explains the presentation of results as interconnected, participant-validated accounts of developmental, professional, and community-based transformation.

### 3.1 RQ 1. How do participants perceive benefits from the mentoring program?

#### 3.1.1 Theme 1.1: benefits of the program perceived by mentees

Mentees described the program as a transformative experience that provided both practical and emotional support. Participation offered guidance on navigating academic and professional challenges, including graduate program applications, funding proposals, and career planning. Mentees also valued learning from mentors with shared backgrounds, interests, or professional trajectories, which made guidance more relatable, actionable, and inspiring. Additionally, access to mentors' professional networks emerged as a significant benefit, opening doors to professors, alumni, and industry contacts and enhancing career opportunities and personal agency.

From these experiences, three interrelated subthemes emerged: 1) Professional Development, where mentors provided guidance, emotional encouragement, and professional advice that supported both personal and academic growth; 2) Learning Through Shared Experiences, in which participants learned from mentors with similar experiences, backgrounds, or career interests, making advice more meaningful and motivating; and 3) Expanding Professional Connections, highlighting how networking opportunities facilitated by mentors helped mentees build relationships critical for academic and career advancement.

According to the organization's evaluation documentation, mentees emphasized the importance of clear communication and proactive engagement. In addition, satisfaction surveys indicated that most mentees identified and worked on 2–3 goals with their mentors, reinforcing the program's effectiveness in goal-oriented development.

Importantly, these subthemes were not experienced in isolation; rather, mentees often described how professional development, shared experiences, and networking opportunities occurred simultaneously within the mentoring relationship.

##### 3.1.1.1 Subtheme 1.1.1: professional development

Participants emphasized how having a mentor provided guidance, practical support, and emotional encouragement throughout their academic and professional development. Mentors helped navigate complex application processes (e.g., graduate programs, funding proposals), build confidence, develop professional skills, and expand networks. This support was instrumental not only for career opportunities but also for personal growth, including overcoming fears and developing leadership and communication abilities.

Participant 2 highlighted the importance of connections, even after having strong academic credentials: “So [my mentor] connected me with a professor. Thanks to my mentor's intervention, they also helped me get closer to him, and there was a really nice synergy because I had just received some funding that I submitted with him.”

Participant 15 described both technical and emotional support during the application process:

“He [mentor] helped me see possibilities, to figure out what exactly I wanted, or to find what I was looking for. He helped me in the process of improving my Letter of Intent for applications, and also improving my CV. One of the things that scared me the most was taking the TOEFL. I feel that my mentor helped me a lot to gain confidence in myself.”

Participant 19 emphasized the long-term relationship and wide range of support received:

“We are still very good friends [mentor and mentee]. He helped me look for universities, and he helped me with the process of writing an email or a cover letter to send to professors. We did mock interviews to know what they might ask me in a master's interview and how to sell myself. He helped me organize my CV. He also helped me with my thesis by giving me tips and advice.”

Finally, Participant 16 reflected on the personal dimension of growth: “I developed several leadership skills that I never thought I had. Honestly, I consider myself a shy and not very extroverted person. But with these opportunities, I think I have developed my ability to express myself quite a bit.”

The AGEAP USA Mentoring Program contributed to mentees' academic and professional development by offering personalized guidance, emotional support, and strategic networking opportunities. Participants highlighted how mentors played a pivotal role in navigating complex processes such as graduate applications and funding proposals, while also fostering personal growth through confidence-building and leadership development. The mentoring relationships extended beyond technical assistance, evolving into long-term connections that empowered mentees to overcome fears and clarify their career paths.

##### 3.1.1.2 Subtheme 1.1.2: learning through shared experiences

Mentees often emphasized the importance of learning directly from mentors whose backgrounds, interests, or career stages mirrored their own. These shared experiences created a strong sense of relatability and offered practical strategies for navigating academic and professional pathways. Participants highlighted how these similarities not only made advice more applicable but also provided reassurance, inspiration, and guidance for future decisions.

Participant 8 reflected on the value of having a mentor with a very similar story: “I was lucky that my mentor's story was very similar to mine. It was more about advice, personal guidance, and perspectives on studying here (…). I even had the chance to meet her in person.” Also, for Participant 14, having a mentor with overlapping academic interests was particularly meaningful: “I felt that when I was doing my thesis, not many people really understood me, or maybe the concepts were too complicated. But [my mentor] was good at explaining things, listening to me, and giving me advice.”

Participant 19 explained how working with a mentor in the same professional field provided encouragement and practical advice:

“I consider myself very young, just an early career, and especially at Central American University, you're just leaving that bubble and about to enter the real world, it's overwhelming. Having someone who has been through it and can say, “Everything will be fine,” even when professors don't respond to emails, was really helpful. My mentor would say, “Don't worry, some people won't answer, but others will. It's okay.” That gave me so much support.”

Similarly, Participant 26 described the mentoring relationship as inspirational because it allowed them to learn from the paths taken by alumni with similar backgrounds: “I think it's a very good tool because it gives you the opportunity to talk with people from the same background, like Alumni of Central American University, who have gone further. It's an inspiration to hear from others how they got where they are.” Finally, Participant 15 emphasized how the program's matching process was key to ensuring meaningful guidance: “I feel like I had a really good match with my mentor. I really liked that when you fill out the form, you indicate your interests, and my mentor was in the same field because he was doing his doctorate.”

The AGEAP USA Mentoring Program's emphasis on matching mentees with mentors who share similar backgrounds, interests, or career stages proved to be a powerful strategy for fostering meaningful guidance. These shared experiences created a strong sense of relatability, enabling mentees to receive practical, emotionally resonant advice.

##### 3.1.1.3 Subtheme 1.1.3: expanding professional connections

Mentees consistently emphasized the pivotal role of networking in shaping their academic and professional opportunities. Through mentors, they gained access to valuable contacts, learned how to approach professors, and built relationships that facilitated career advancement. These connections not only provided practical advantages but also fostered confidence, guidance, and trust during critical transitions.

Participant 10 highlighted how a mentor's experience and advice made navigating new opportunities much easier: “The mentor I was assigned has many years of experience working here. Her advice on how to approach professors and request another program helped me extend my stay and secure a new project”. Similarly, Participant 2 emphasized how networking created access to professors who might otherwise have been out of reach: “Just getting a professor to open the door for you, to reply to your email, that is the most beneficial thing one can imagine. Networking makes the professor respond, and that gets you to the interview.”

For Participant 3, mentors played the role of professional guides, offering encouragement and direction: “Part of networking is getting to know people you never interacted with before. Having someone more experienced serve as that older brother in the professional sphere helps guide you—if you allow yourself to be helped.”

Participant 26 described how networking through a mentor provided direct links to alumni with similar backgrounds: “He [mentor] gave me the contact of another Central American University alumnus… he could share his experience and help me think through future career decisions. That perspective was very helpful as I considered my options.”

In some cases, mentors facilitated connections with researchers who were directly relevant to academic work. Participant 14 shared: “My mentor gave me the WhatsApp contact of a researcher whose paper I was citing for my thesis. That first approach gave me the chance to ask questions directly and build a link with the lab where I later worked.”

Finally, Participant 19 emphasized how networking through a mentor made the process of contacting professors abroad more effective: “I asked my mentor about a professor at Nebraska, and he said, “I know her from conferences, I'll write to her for you.” That helped her feel more secure in considering me, which made the whole process much easier.”

Together, these accounts highlight networking as a key outcome of mentoring—a process that not only opened doors to new opportunities but also offered reassurance, guidance, and tangible pathways for professional growth.

#### 3.1.2 Theme 1.2: benefits of the program perceived by mentors

Mentors reported that participation fostered their own professional and personal growth. Through mentoring, they enhanced communication, leadership, and empathy, learning to navigate conversations with tact and to adapt to different mentee needs. Mentors emphasized the importance of role modeling, demonstrating authentic leadership through personal experiences and lessons learned from successes and mistakes.

The program also promoted continuous learning for mentors. They gained exposure to younger generations' perspectives, developed technological competencies, and refined organizational strategies. Mentoring became a two-way exchange, where mentors not only supported mentees but also expanded their own knowledge, adaptability, and professional capabilities. Three related subthemes complemented the theme: 1) Communication Skills: Mentors practiced and improved effective, assertive, and empathetic communication while guiding mentees. 2) Role Modeling: Mentors shared authentic experiences and led by example to influence mentees' decisions and development. 3) Continuous Learning: Mentors gained new skills, technological knowledge, and insights from mentees, fostering ongoing personal and professional growth.

From the organization's evaluation documentation, mentors appreciated the opportunity to contribute to mentees' growth while reflecting on their own journeys. Mentors suggested aligning mentor-mentee pairs based on future interests (e.g., academia vs. industry) to enhance relevance and impact.

##### 3.1.2.1 Subtheme 1.2.1: communication skills

The most frequently highlighted skill developed through the mentoring program was communication, expressed in multiple forms. Participants reflected on learning effective and assertive communication, active listening, empathy, and the ability to adapt conversations to different contexts, generations, and needs. These experiences underscored how mentoring relationships provided opportunities to practice and refine communication in both professional and personal settings.

Participant 3 described how mentoring others strengthened their ability to express ideas concisely and effectively:

“I think communication, because I practiced it while reviewing the letters of the people I mentored. That also helped me practice effective communication, as well as learning to summarize things. When you practice and review, you learn how to compile necessary information into short paragraphs.”

For Participant 11, effective communication was central not only to leadership but also to building relationships across generations: “I think effective communication is important. You learn, one way or another, how to deal with different generations. That is essential for leadership: having effective communication.” Participant 17 emphasized the importance of empathy and responsibility in moments requiring sensitivity: “I don't know if I would call it assertive communication, because it feels deeper than that. It's a kind of communication where you have to navigate carefully.”

From another perspective, Participant 18 reflected on transitioning from being a mentee to becoming a mentor and how that shift required assertive and proactive communication: “The program helped me make that switch because being called a mentor officially meant I had to provide what I once expected from a mentor. That required better communication and planning.” Also, Participant 25 highlighted the broader role of communication in mentoring, both technically and personally:

“One of the most important skills is learning to communicate aspects you don't usually practice, such as giving guidance or advice. It's about sharing from your own experience and creating scenarios for mentees that reflect what they may face.”

Finally, Participant 26 explained how the mentoring program helped them realize the importance of listening as an integral part of communication: “I learned to improve communication and listening, which is harder than it seems. Sometimes you hear but don't truly interpret what the other person is saying. That was a key lesson, along with time management.”

Together, these reflections illustrate how mentoring provided a practical and transformative space for participants to develop communication skills, not only as a professional competency but also as a leadership quality rooted in empathy, clarity, and connection.

##### 3.1.2.2 Subtheme 1.2.2: role modeling

Mentors emphasized their role as models of leadership, guiding mentees not only through advice and tools but also through their own leadership by example. They reflected on how their own experiences, successes, and mistakes helped mentees navigate decisions with greater clarity. By sharing authentic stories, offering strategies to reduce stress, and modeling resilience, mentors influenced mentees' academic, professional, and personal growth.

Participant 4 described role modeling as rooted in authenticity and action rather than words alone: “Much of mentoring is about examining your own career, how you've behaved. I think the best way leadership is demonstrated is through action, by doing things.” Similarly, Participant 13 reflected on influencing mentees by sharing the same strategies that once supported their own success:

“I was sharing the tools that worked for me throughout my professional and personal journey. For example, with one mentee who wasn't sure what to do, I told her, “Sign up for the GRE; surely your university offers practice options, and you can take the exam there.” I tried to influence her to take actions that had helped me.”

For Participant 16, role modeling meant offering direction and helping mentees avoid unnecessary stress: “I would say it's about instructing someone in the right way, helping the mentee not to stress too much, and not letting them stray too far from what's really needed to complete the process they want.”

Finally, Participant 25 emphasized the importance of being a model that mentees could relate to and learn from: “It's also about serving as a model, seeing yourself as someone they can reflect on, someone who has already gone through things they will likely face in their future path.”

Together, these accounts highlight how mentors saw themselves as role models, using their lived experiences and authentic leadership to guide mentees, reduce uncertainty, and inspire positive decision-making.

##### 3.1.2.3 Subtheme 1.2.3: continuous learning

Participation in the program strengthened mentors' organizational skills and adaptability, particularly in time management, meeting planning, and proactivity. Mentors also reported learning from younger generations, gaining exposure to technology, and appreciating new perspectives, highlighting that the mentoring process fostered a two-way exchange of knowledge and growth.

Participant 1 reflected on the intergenerational learning experience:

“I am from an older generation. When we were at university, there was no technology, no computers, no internet, no phones. There is a big gap between generations. I have learned a lot about why everyone younger than me works the way they do, how they use technology, and how they have developed. For me, seeing things I never had the opportunity to experience has also benefited me greatly.”

Similarly, Participant 12 emphasized gaining insight from mentees' perspectives: “I learned from the perspectives of the younger participants and the goals they want to achieve, whether in continuing studies here or in their professional life in the United States.” Also, participant 23 highlighted practical skills acquired through mentoring younger generations: “Surprisingly, I learned how to conduct interviews properly and how to develop myself on social media. I also learned to manage technology better, like hosting meetings on Zoom, which is important for someone from my generation.”

Together, these reflections demonstrate that mentors not only contributed to the development of mentees but also engaged in continuous learning by themselves, gaining new skills, broadening their perspectives, and adapting to intergenerational and technological changes.

#### 3.1.3 Theme 1.3: benefits of the program perceived by organizers

Organizers and volunteers highlighted how participation strengthened their leadership, organizational, and coordination abilities. Managing teams, planning meetings, and navigating complex schedules provided practical experience in program administration.

Beyond technical skills, involvement fostered connected growth. Participants expanded professional networks, reconnected with the AGEAP USA community, and contributed to the collective identity of the alumni network. Organizers developed leadership, communication, and time management skills, enhancing their capacity to facilitate projects, support mentees, and assume responsibility in collaborative settings. Two related subthemes emerged: 1) Organizational Skills Development: Participants improved their ability to coordinate teams, plan agendas, and manage complex group dynamics, and 2) Connected Growth: Engagement promoted professional networking, community reconnection, and reinforcement of collective alumni identity.

Additionally, seven of the interviewees previously participated in the program as mentors or mentees and later served as organizers. Organizers described this continuity as a meaningful outcome of the program, noting that their prior participation deepened their understanding of the program's goals and strengthened their commitment to supporting new cohorts.

From the evaluation documentation provided by the organization, organizers suggested clearer communication protocols and expectations at the start of the program to improve coordination. In addition, there was a strong emphasis on maintaining responsiveness and accountability, such as following up with participants who hadn't responded. The experience of managing mentoring logistics helped volunteers develop transferable skills applicable to other professional settings.

##### 3.1.3.1 Subtheme 1.3.1: organizational skills development

Volunteers and program organizers highlighted learning how to coordinate people, manage time, and lead teams in complex contexts. Participation in these roles offered practical experience in organizing meetings, planning agendas, and navigating diverse schedules and team dynamics, fostering both personal and professional growth.

Participant 7 reflected on learning to coordinate teams and plan meetings effectively: “I first learned a lot about what was happening within the organization itself. I used to think they just got together, but seeing the structure, ‘let's get organized, I benefited from learning how to organize teams and meetings.” Similarly, Participant 5 emphasized connecting mentees to opportunities and building networks: “I mostly worked with Central American University students in their senior year who were interested in graduate programs, supporting tasks like reviewing emails before sending them to professors. I connected them with colleagues or people in my network.” Also, Participant 9 highlighted learning from program successes and challenges: “I learned to identify what works and what doesn't in programs like this. That was a great learning experience because I had never been involved in something like it before.”

Together, these accounts illustrate how volunteering and organizing roles in the mentoring program helped participants develop organizational, leadership, and coordination skills while gaining practical insight into effective program management.

##### 3.1.3.2 Subtheme 1.3.2: connected growth

Participation in the program allowed alumni to expand professional networks, reconnect with the AGEAP USA community, and strengthen a sense of collective identity. Through involvement as mentors, coordinators, or organizers, participants developed leadership, communication, and organizational skills while reestablishing ties with peers and the broader alumni network.

Participant 11 emphasized reconnecting with the community and developing a sense of responsibility:

“As an organizer, the most important thing was meeting people. It's not so much what you can do, but what the people you meet know you can do. Reconnecting with the Central American University community was very important to me. I hadn't been involved in anything Central American University -related for about 8 years, so regaining that sense of belonging was significant. Staying active in conversations and opportunities was important too.”

Participant 20 highlighted networking and skill development: “I perceived networking with other coordinators since I didn't know them before. I also developed leadership and communication skills. These were not tangible things *per se*, but skills that improved through participation.” In addition, Participant 12 reflected on mentorship and career advantages: “Among the main benefits, I also had a mentor. I learned a lot about private sector experience from my mentor. Another benefit was including this experience on my CV, which was very well-received in some job interviews.” Lastly, Participant 18 noted more minor benefits but recognized value in visibility: “I didn't perceive many benefits, maybe just being more informed about what was happening in the mentoring program. Also, I could include it on my CV.”

These accounts illustrate how participation fostered connected growth, combining professional networking, community reconnection, and skill development while contributing to the collective identity of the Central American University alumni network.

### 3.2 RQ 2. What challenges did participants encounter during their involvement in the mentoring program?

The challenges identified in the AGEAP USA Mentoring Program reflect broader issues in volunteer-based mentoring initiatives, including engagement gaps, structural limitations, and communication barriers. While many mentors found the experience rewarding, the program's effectiveness was often hindered by mismatched expectations, lack of structure, and inconsistent participation.

According to the organization's evaluation documentation, participants in the mentoring program reported encountering challenges that affected their experience and program outcomes. First, engagement and responsiveness were inconsistent, with some mentors and mentees unsure who should initiate contact, resulting in missed opportunities for connection. Second, mismatched expectations created confusion, as some participants anticipated outcomes such as internships or graduate school placements that were not the program's primary goals.

These challenges were grouped into three main themes: (1) lack of commitment by mentees, (2) difficulties in establishing connections, and (3) challenges in recruiting mentors and maintaining their commitment.

#### 3.2.1 Theme 2.1: lack of commitment by mentees

A recurring theme among mentors was the low level of engagement and initiative from mentees. Many mentors reported that mentees failed to follow up after initial meetings, did not respond to messages, or abandoned the program altogether.

Participant 13 said, “I don't think it was the best experience... I had a single meeting with both of my mentees and then never heard back,” which was echoed by participant 28, “I used to tell them, let's meet... but they never sent me anything. Then there comes a point where you feel like you're asking for a favor.” In addition, participant 20 stated that “Many mentees lose interest or do not respond to emails on time.”

This disengagement disrupted the mentoring relationship and led mentors to question the value of their involvement. Participant 26 reflected, “At some point, it felt like they were doing me a favor by replying.” Similarly, Participant 17 noted, “I stopped joining other mentoring programs to prioritize this one, but it felt like a wasted year because my mentees never followed through.” The lack of reciprocity and initiative from mentees created emotional fatigue among mentors, many of whom had volunteered with the intention of giving back to the AGEAP USA community. As Participant 25 put it, “At first I thought I was the problem, but I realized it's the process that doesn't work for me.” These sentiments suggest that the program's structure may need to better support mentors in navigating inconsistent engagement and provide clearer expectations for mentees from the outset.

These sentiments point to deeper structural issues within the program. Mentors' experiences suggest that clearer guidance and better support systems are needed to help them navigate inconsistent engagement and to establish shared expectations from the outset. Moreover, this pattern aligns closely with findings from the AGEAP USA census, where many who chose not to participate in the mentoring program cited time constraints, unclear expectations, and limited communication as their main deterrents. The convergence between mentors' lived experiences and non-participants' perceptions highlights how organizational challenges, rather than individual attitudes, may undermine both participation and retention. In essence, the same systemic barriers that discouraged some from joining also contributed to the disengagement of those who did.

#### 3.2.2 Theme 2.2: difficulties in establishing connections

Mentors highlighted the challenge of building rapport with mentees, especially when there was a mismatch in interests, professional background, or communication style. For example, participant 25 mentioned, “We couldn't create that connection... I don't think it's the fault of either the mentee or mine, but rather the type of mentoring,” which was echoed by participant 18, mentioning that “My mentee asked me if I had a doctorate... I felt that he was disappointed. Maybe there simply wasn't compatibility.” Some mentors suggested that mentees were not emotionally or professionally ready to engage in meaningful mentoring relationships, especially during transitional academic phases.

In addition, organizers and mentors alike faced logistical difficulties in establishing connections in terms of coordinating meetings, managing time zones, and balancing volunteer responsibilities with professional obligations. Participant 3 mentioned, “The schedules do not match... There are people who take a long time to respond or never respond,” and participant 12 said, “The times we had to meet often didn't work out.” Therefore, the lack of centralized scheduling tools and formal accountability mechanisms further complicated coordination efforts.

#### 3.2.3 Theme 2.3: difficulty recruiting mentors and maintaining commitment

Organizers reported challenges in recruiting and retaining mentors, especially those with relevant experience or availability. The imbalance between mentee demand and mentor supply was a persistent issue. Participant 1 stated that “We had more mentees than mentors. That was a big challenge.” This shortage of mentors often led to mismatches in expertise and limited the ability to personalize mentoring relationships. Participant 20 highlighted the instability in the organizing team, noting: “The team that is now is completely new and there is nobody from last year.” Such turnover disrupted continuity and placed additional pressure on incoming volunteers to rebuild institutional knowledge and processes from scratch. Additionally, some mentors expressed frustration over mentees' lack of seriousness, which discouraged them from continuing in future cycles.

### 3.3 RQ3. What motivated Central American University alumni to participate in the mentoring program from 2020 to 2024?

From the evaluation documentation provided by the organization, research memos, and interviews, participants were motivated to join the mentoring program by a desire to support others, foster professional development, and strengthen ties within the AGEAP USA community. Mentors expressed satisfaction with their role and felt prepared to guide mentees, as reflected in the mid-year evaluation responses. Organizers and volunteers were driven by the opportunity to contribute meaningfully, reconnect with alumni, and promote shared values, as emphasized in the program suggestions. Mentees were motivated by the chance to work on specific goals, such as academic planning and career guidance, many of which they successfully identified and pursued during the program, according to questionnaire feedback. Two primary themes emerged: Mentees' Motivations and Mentors and Organizers' Motivations.

Mentees' motivations encompassed three subthemes: seeking guidance on professional opportunities, learning from real and shared experiences, and overcoming fears and uncertainties related to academic and career transitions. Motivations among mentors and organizers likewise comprised three subthemes: contributing to the AGEAP USA community, improving transition experiences for future alumni, and strengthening a sense of belonging with AGEAP USA.

#### 3.3.1 Theme 3.1: mentees' motivations

Mentees who participated in the AGEAP USA Mentoring Program expressed a range of motivations primarily shaped by their academic and professional aspirations, as well as the challenges associated with navigating transitions in unfamiliar systems. Their participation was driven by a desire for guidance, learning from alumni experiences, and emotional support during periods of uncertainty.

##### 3.3.1.1 Subtheme 3.1.1: seek guidance for professional opportunities

A primary motivator for mentees was the desire for guidance on academic and professional opportunities, particularly internships and graduate school applications. Many mentees were navigating complex decisions and saw the program as a strategic resource. Participant 12 shared that their motivation stemmed from a clear academic goal: “I knew that I was interested in doing it (grad school) here in the United States. So, it was a way for me to say, okay, I can talk to someone who has already gone through the process.” This sentiment was echoed by Participant 15, who expressed anxiety about standardized testing and found reassurance through their mentor: “One of the things that scared me the most was taking the TOEFL. My mentor helped me a lot to gain that confidence.” Participant 2 also emphasized how the program helped them feel closer to their long-term academic aspirations: “I feel a little closer (to the PhD program) thanks to the things we have done with the program.”

Beyond this support, mentees frequently sought practical assistance with the technical aspects of academic applications. This included help with crafting CVs, writing letters of intent, preparing for interviews, and understanding the nuances of application timelines and requirements. Mentors were often seen as gatekeepers of insider knowledge, as individuals who had successfully navigated the same systems and could offer tailored advice based on lived experience.

For example, participant 9 described how their mentor helped them organize their CV and prepare for graduate school interviews: “He helped me organize my CV... we did mock interviews to know what they might ask me in a master's interview.” Mentors also provided direct connections to professors, research opportunities, or institutional contacts, which mentees viewed as invaluable. Participant 2 noted: “Just getting the professor to respond to your email is the most beneficial thing one can imagine.” These examples illustrate that mentees were seeking general advice and actively looking for mentorship that could translate into tangible academic and professional outcomes. The program served as a bridge between aspiration and action, helping mentees move from uncertainty to informed decision-making.

##### 3.3.1.2 Subtheme 3.1.2: to learn about real experiences

Mentees were motivated by the opportunity to hear personal stories from Central American University alumni who had successfully transitioned into graduate programs or professional roles. These real-life experiences provided mentees with practical insights, helping them visualize their own goals. Participant 8 expressed a clear desire to connect with alumni who had already navigated the complexities of professional life abroad: “I wanted to meet Alumni of Central American University who are already in the industry, to learn from them, especially on topics like paperwork.” This previous quote highlights the mentee's interest in learning career trajectories and the bureaucratic and logistical aspects of working internationally. Participant 10 emphasized the value of shared experience and personal alignment with their mentor: “The story of my mentor was very similar to mine, so she gave me personal guidance and study perspectives.” Participant 30 reflected on the inspirational aspect of hearing success stories: “Listening to others, how they have managed to get to where they are, has been an inspiration in my personal development.”

For mentees, these interactions helped explain the process of transitioning into graduate school or the workforce. Mentors' support also served as a source of motivation, reinforcing the belief that success was attainable and that others from similar backgrounds had already paved the way. Moreover, this peer-based learning was especially valuable for mentees who felt disconnected from formal institutional support or lacked access to professional networks. The mentoring program filled an important gap by offering informal, yet impactful guidance rooted in real-world experience.

##### 3.3.1.3 Subtheme 3.1.3: overcome fears and uncertainties

Beyond academic and professional guidance, many mentees joined the AGEAP USA Mentoring Program seeking emotional support to navigate the psychological challenges of transitioning into graduate school or the workforce. These mentees often expressed feelings of fear, doubt, and uncertainty about their future, particularly regarding their preparedness, language barriers, and career direction. Participant 14 shared a moment of vulnerability, stating, “I wasn't sure about the career I had chosen. My mentor encouraged me to continue.” This quote reflects how mentorship helped mentees reframe their self-doubt and regain confidence in their academic and professional choices.

The mentor's encouragement served as a stabilizing force during a period of uncertainty. Participant 9 echoed this sentiment, describing how their mentor helped shift their mindset: “I had many questions... I felt like I might not be capable. But my mentor made me see that I wasn't that far off.” In addition, Participant 15 highlighted a specific fear related to standardized testing: “One of the things that scared me the most was taking the TOEFL. My mentor helped me a lot to gain that confidence.”

For mentees like this, the mentoring relationship extended beyond academic advice; it became a source of emotional resilience. Mentors helped mentees confront their fears, whether related to language proficiency, application processes, or broader career uncertainties. Also, one important thing to highlight is that the mentoring program provided a safe space for mentees to express their concerns, receive personalized feedback, and build the confidence needed to take the next steps in their academic and professional journeys.

#### 3.3.2 Theme 3.2: mentors and organizers' motivations

Mentors and organizers who participated in this research were involved in the AGEAP USA Mentoring Program from 2020 to 2024 and expressed a range of motivations rooted in personal experience, community values, and a desire to strengthen institutional ties. Their participation was often driven by a sense of responsibility, reflection on their own academic journeys, and a commitment to support future generations of Central American University alumni.

##### 3.3.2.1 Subtheme 3.2.1: contribute to the AGEAP USA community

A strong and recurring motivator among mentors and organizers was the desire to give back to the AGEAP USA community. Many participants had benefited from informal mentorship or support during their own transitions and saw the program as an opportunity to pay it forward.

Participant 24 reflected on this sentiment, stating: “I was motivated by the idea of being able to help the new classes. I would have liked to have had that experience when I graduated.” This quote highlights a retrospective awareness of missed opportunities and a desire to create a better experience for future cohorts. Similarly, Participant 20 emphasized the nature of his motivation: “The fact that I could help more people from Central American University.” This framing suggests that the mentoring program was not just about individual relationships, but about strengthening the broader AGEAP USA network and culture of support. Participant 11 reinforced this idea by recognizing the value of institutionalizing mentorship: “I thought it was an excellent idea to have a mentoring group that can guide them.”

This motivation also carried a sense of pride and responsibility. Mentors viewed their role as stewards of knowledge and experience, and organizers saw themselves as facilitators of a community-driven initiative. Together, they contributed to a mentoring culture that emphasized academic success and personal growth, professional development, and the strengthening of Central American University identity across generations.

##### 3.3.2.2 Subtheme 3.2.2: improve transition experience

Mentors and organizers were motivated by a desire to improve the transition process for younger Central American University alumni, from undergraduate studies to graduate school, internships, or professional careers. This motivation was rooted in personal reflections on the challenges they themselves had faced during similar transitions. Mentors expressed that they had struggled with a lack of guidance, limited access to resources, or confusion about how to navigate complex systems such as graduate admissions, visa applications, or job searches.

Participant 7 shared his own experience: “I had a super bad experience applying for internships. My idea was to help the third-year undergraduate students prepare their applications.” This quote reflects a proactive effort to transform a personal challenge into a collective solution. By joining the mentoring program, participants aimed to prevent others from repeating the same mistakes and to provide the kind of support they had lacked. Participant 9 echoed this sentiment, emphasizing the importance of recognizing privilege and extending support to those who may not have had the same opportunities: “Just because I have had that fortune does not mean it is the reality for everyone. That motivated me to support and promote this type of transition.”

Mentors and organizers understand that not all students have access to professional networks, insider knowledge, or the confidence to pursue opportunities abroad. Their involvement in the program was driven by a commitment to fill those gaps and make the transition process more inclusive and accessible. Mentors and organizers see the AGEAP USA Mentoring Program as a tool to institutionalize support, and their goal is to help individual mentees and strengthen the overall infrastructure of transition for Central American University alumni, ensuring that future generations will be better equipped to pursue their academic and professional goals.

##### 3.3.2.3 Subtheme 3.2.3: strengthen the sense of belonging with AGEAP USA

Participating in the AGEAP USA Mentoring Program was a professional and altruistic endeavor, and it was also a personal experience that helped them reconnect with their Central American University identity. After years of academic and professional growth, often in international contexts, some Central American University alumni found themselves distanced from the institution and its community. The mentoring program offered a meaningful way to re-engage AGEAP USA, reaffirm shared values, and contribute to a legacy they still felt part of.

Participant 9 described this reconnection as a powerful emotional experience: “Recovering that sense of belonging was something important.” This quote reflects how involvement in the program helped revive a sense of institutional pride and cultural identity. For alumni who had been disconnected from Central American University for years, mentoring became a way back to their roots. Participant 7 emphasized how the program facilitated a more formal and structured involvement with the AGEAP USA community: “It helped me learn how things are organized and to get more formally involved with the AGEAP USA community.” This sense of belonging was shown in active participation, leadership, and a commitment to the program's goals.

This mentoring program allows alumni to transform their individual experiences into community contributions, reinforcing the idea that Central American University is not just a university; it is also a lifelong network and identity. This emotional connection strengthened their dedication to mentoring and organizing, making their participation more than just a task; it became a reaffirmation of who they are and where they come from.

### 3.4 RQ4. To what extent did the AGEAP USA mentoring program achieve its stated objectives from 2020 to 2024?

This research question examined the degree to which the program met its four stated program objectives: (1) increasing professional relationships among alumni in the U.S., (2) supporting alumni applying to graduate programs, (3) assisting alumni with job applications, and (4) creating opportunities for alumni to share experiences and develop mentoring and leadership skills.

Regarding the first program objective, participants reported expanded networks, often gaining access to professors, researchers, and alumni with shared disciplinary or professional backgrounds. Mentees described networking as a valuable aspect of the program, frequently crediting mentors for connecting them with new academic or industry contacts. These findings indicate strong progress toward this objective, though networking outcomes varied with mentee engagement levels.

Mentees reported being introduced to professors, researchers, and alumni in their fields. For example, from the stated themes merged from the previous research questions, participant 2 described being directly connected to a graduate professor whose collaboration later supported a funded project, and participant 14 noted receiving the WhatsApp contact of a researcher whose work they cited in their thesis, an interaction that led to lab-based collaboration (see Subtheme 1.1.3: expanding professional connections). The internal census, as part of the triangulation evidence, further supported these findings, documenting that 39 respondents had participated in the program and engaged in alumni activities, reinforcing network expansion across cohorts.

Regarding the second program objective, mentees reported substantial guidance on CV development, statements of purpose, program selection, funding strategies, and communication with potential advisors. Mentors frequently engaged in reviewing application materials, conducting mock interviews, and helping mentees navigate uncertainty about graduate pathways. Mentees attributed their admission success or improved preparedness to mentor support, demonstrating clear achievement of this objective.

Data provide evidence that the program supported graduate school preparation. For example, from the stated themes merged from the previous research questions, participant 15 described gaining confidence to take the TOEFL after mentor support, while participant 9 highlighted how mock interviews helped them prepare for university admissions. Other participants attributed successful applications, increased preparedness, or clearer academic direction directly to their mentors' guidance (see Subtheme 1.1.1: professional development).

Answering the third program objective, participants reported receiving support with résumé and cover letter revisions, interview preparation, and strategic advice for contacting employers or navigating industry expectations in the United States. While fewer mentees engaged in job-focused mentoring than in graduate-school support, those who did reported meaningful benefits that improved their confidence and prepared them to enter the workforce.

While fewer participants sought job-related support than graduate assistance, those who did reported impactful outcomes. For example, from the stated themes merged from the previous research questions, participant 10 described receiving guidance on how to approach potential employers and secure a new project extension. Participant 3 emphasized learning how to reach out professionally to new contacts, describing mentorship as a professional “older brother” who guided them through unfamiliar workplace expectations. These examples suggest partial but positive fulfillment of the objective, influenced by mentees' career stage and readiness (see Subtheme 1.1.3: expanding professional connections).

Lastly, in response to the fourth program objective, mentors reported developing communication, empathy, leadership, and organizational skills through their participation. Mentors and organizers described mentoring as a reflective experience that enhanced their leadership identity and confidence. Central American University alumni also engaged in multiple roles, such as mentor, mentee, and organizer, deepening opportunities to share experiences and shape the mentoring culture across program cohorts.

Participant 11 described mastering cross-generational communication, participant 25 highlighted growth in assertive communication, and participant 16 reported significant development in leadership and confidence. In addition, seven participants served as organizers after being mentors or mentees, an indication of leadership development and sustained commitment to the program. Organizers described improving their coordination, time management, and team leadership, while strengthening their sense of belonging within the alumni community. These findings indicate that the program fully achieved this objective (see Subtheme 1.2.1: communication skills).

Finally, across all four objectives, the analysis shows that the program achieved both the intended outcomes and additional emergent outcomes valued by participants, such as emotional support, belonging, and intergenerational learning.

## 4 Discussion

The interpretation of the findings is grounded in the methodological foundations of Outcome Harvesting, which emphasize participant-generated evidence of change and the retrospective analysis of contributions. Following Phase 5 of the methodology, the outcomes identified in this evaluation were interpreted through theoretical frameworks, particularly relational mentoring and communities of practice (Ataizi, 2012; Lave and Wenger, 1991; Ragins, 2012). This approach is consistent with Outcome Harvesting's requirement that evaluators situate substantiated outcomes within broader conceptual and practical contexts (Wilson-Grau and Britt, 2012). Rather than limiting interpretation to whether the program met predefined objectives, this section examines the full spectrum of intended and emergent outcomes, including professional development, leadership growth, strengthened alumni identity, and expanded networks, that participants attributed to their involvement in the program. Such interpretation is supported by the literature, which shows that mentoring programs often generate relational, identity-based, and community-oriented changes that exceed formal objectives (Crisp and Cruz, 2009; Montgomery and Page, 2025).

### 4.1 Benefits for mentees

Mentees consistently described the program as transformative, citing professional development, learning through shared experiences, and expanding professional connections as key outcomes. These findings align with the mentoring literature, which emphasizes the importance of emotional support, career guidance, and network building in effective mentoring relationships.

For instance, mentees reported gaining confidence in navigating graduate applications and overcoming fears related to standardized testing and academic transitions. This echoes findings by Al-Aqeel and Alhumaid (2024), who noted that mentoring programs significantly enhance mentees' self-efficacy and academic preparedness. Similarly, mentors‘ role in facilitating access to professional networks and insider knowledge underscores the value of mentoring as a bridge between aspiration and action (Crisp and Cruz, 2009).

The importance of shared backgrounds and relatable experiences was also emphasized, supporting the idea that cultural and experiential alignment enhances mentoring effectiveness. Patel et al. (2024) argue that culturally responsive mentoring is crucial for international students, as it fosters trust and relevance in guidance.

### 4.2 Benefits for mentors and organizers

Mentors reported growth in communication skills, role modeling, and continuous learning. These outcomes suggest that mentoring is not a one-way process but a reciprocal relationship that benefits both parties. Mentors developed empathy, leadership, and adaptability, skills that are central to professional development.

This aligns with findings by McHugh and Gruppen (2021), who highlight that mentors often refine their interpersonal and pedagogical skills through mentoring engagements. Moreover, mentors' reflections on learning from younger generations and adapting to new technologies support the concept of intergenerational learning, as discussed by Moloney et al. (2023).

The role-modeling aspect, in which mentors share authentic experiences and guide mentees through real-world challenges, reinforces the importance of experiential learning in mentoring (Salim, 2021; Speizer, 1981). Organizers and volunteers highlighted improvements in organizational skills and connected growth. Their involvement facilitated leadership development, team coordination, and a renewed sense of belonging within the Central American University alumni network.

This mirrors Rhodes (2005) findings, which emphasized that mentoring programs can strengthen community identity and foster collective engagement. The program's ability to reconnect alumni and promote institutional pride suggests that mentoring initiatives can serve as tools for community building and legacy reinforcement.

### 4.3 Challenges in program implementation

The AGEAP USA Mentoring Program demonstrates strong potential to support Central American University alumni in their academic and professional transitions. However, addressing structural and engagement-related challenges is essential for its sustainability. The evaluation of this mentoring program revealed a complex landscape of challenges and motivations that shaped participant experiences. These findings align with broader patterns in the mentoring literature, particularly in volunteer-based and cross-cultural mentoring contexts (Crisp and Cruz, 2009; Eby et al., 2008; Sambunjak et al., 2006).

#### 4.3.1 Mentee engagement and commitment

One of the most significant challenges was mentees' lack of commitment, which disrupted mentoring relationships and led to emotional fatigue among mentors. Reports of missed meetings, unresponsiveness, and disengagement were common, echoing concerns in mentoring research about the importance of mentee readiness and sustained engagement (Allen et al., 2006; Eller et al., 2014). Mentors expressed frustration over the absence of follow-through, which undermined their motivation and raised questions about the program's structure and expectations.

#### 4.3.2 Mentor–mentee compatibility and relationship-building

Another challenge was the difficulty in establishing meaningful connections. Mismatches in professional interests, communication styles, and emotional readiness hindered the establishment of relationships. This aligns with the existing literature, which emphasizes the importance of compatibility and shared goals in mentoring relationships (Ragins and Kram, 2007; Straus et al., 2013). Barriers such as time zone differences and scheduling conflicts further complicated interactions, especially in a volunteer-driven program without centralized coordination tools. Additionally, the program faced recruitment and retention issues, particularly in securing mentors with relevant experience and availability.

Organizers noted an imbalance between mentee demand and mentor supply, as well as high volunteer turnover. These issues mirror findings from mentoring studies that highlight the need for institutional support, recognition, and continuity to sustain volunteer engagement (Eby et al., 2013; Rhodes, 2005).

### 4.4 Motivations for participation

Despite these challenges, participants were motivated by a strong desire for professional guidance. Many mentees sought help navigating graduate school applications, internships, and job searches. Mentors were seen as gatekeepers of insider knowledge, offering tailored advice based on lived experience. This reflects the value of mentoring as a bridge between aspiration and action, particularly for international students facing unfamiliar systems (Patel et al., 2024; Wilczewski and Alon, 2023).

Mentees also expressed a desire to learn from real experiences, finding inspiration in mentors' personal stories. These narratives helped mentees visualize their own goals and overcome fears related to academic transitions, language barriers, and career uncertainty. Emotional support emerged as a key theme, with mentors helping mentees reframe self-doubt and build confidence, an outcome widely supported in mentoring literature (Eller et al., 2014; Sambunjak et al., 2006).

Mentors and organizers were primarily motivated by a desire to give back to the Central American University community, improve the transition experience for younger alumni, and strengthen their sense of belonging. Many reflected on their own struggles and saw the program as a way to institutionalize support and foster intergenerational solidarity. This aligns with research on mentoring as a tool for community building and identity reinforcement (Allen et al., 2006; Rhodes, 2005).

### 4.5 Accomplishment of program objectives

The strengthened evidence for RQ4 demonstrates that the AGEAP USA Mentoring Program achieved substantial progress toward all four stated objectives and generated additional emergent outcomes consistent with relational mentoring and communities-of-practice frameworks. Participants' accounts of gaining access to academic advisors, receiving tailored graduate school guidance, practicing interview skills, and strengthening professional networks directly reflect intended program goals and align with literature emphasizing the role of mentoring in facilitating academic transitions and career preparedness (Crisp and Cruz, 2009; McHugh and Gruppen, 2021). The development of leadership and communication skills among mentors and organizers further supports relational mentoring theory, which highlights the reciprocal nature of growth within high-quality mentoring relationships (Ragins, 2016). The fact that seven participants moved into organizer roles after serving as mentees or mentors suggests the formation of a sustainable community of practice, where alumni gradually assume more central roles and contribute to the continuity of shared professional and cultural knowledge (Lave and Wenger, 1991).

### 4.6 Limitations of the evaluation

The main limitation of this evaluation was the sole focus on primarily qualitative evidence, relying on interviews and document analysis. While rich in detail, the absence of quantitative metrics limits the generalizability of findings. Future evaluations could benefit from integrating surveys or other quantitative tools to strengthen data triangulation and provide more robust indicators of success (Saldaña, 2016; Wilson-Grau and Britt, 2012). Additionally, the program's annual turnover of participants complicates longitudinal assessment, as each cycle involves new individuals with varying levels of engagement and experience.

The study relied on a purposeful qualitative sample of 30 participants, which, while appropriate for Outcome Harvesting and sufficient to reach meaning saturation across heterogeneous roles, limits the generalizability of results. Participants who volunteered for interviews may have been more engaged and reflective than those who did not. In addition, although the sample intentionally included mentees, mentors, and organizers across multiple cohorts, not all participant experiences were represented equally. Some cohorts and roles were more visible than others, which may have shaped the prominence of certain outcomes while underrepresenting less successful mentoring experiences.

While the Outcome Harvesting approach is well-suited to capturing emergent, relational, and unintended changes, it does not assess causal impact or quantify the magnitude of change. Alternative approaches, such as mixed-methods designs, pre-post surveys, or comparison groups, could strengthen future evaluations by complementing harvested outcomes with standardized measures of satisfaction, engagement, or career progression.

The wide range of participant backgrounds, career stages, and engagement levels, while a strength for capturing diverse perspectives, led to heterogeneous mentoring experiences. Outcomes varied depending on participants' readiness, expectations, and availability, making it difficult to draw uniform conclusions about program effectiveness. These contextual differences underscore the importance of interpreting outcomes as context-specific contributions rather than standardized program effects.

### 4.7 Lessons for Mentoring Programs: Applying Relational and CoP Frameworks

Our findings reinforce relational mentoring as a viable design anchor: mentoring relationships that center mutuality, trust, and reciprocity enable psychological safety, realistic problem-solving, and bidirectional learning rather than one-way guidance, an interpretation consistent with recent evidence framing mentorship as reciprocal growth that transcends hierarchical models in academic development contexts (Hansen et al., 2025). In parallel, CoP clarified how structured participation (e.g., repeated interactions around authentic tasks, access to tacit knowledge, and norm-interpreting conversations) moves participants along a trajectory from legitimate peripheral participation toward fuller engagement in academic and professional communities (Adkisson et al., 2020; Holland, 2018). In addition, emerging research grounded in social exchange theory shows that relational quality and perceived organizational support shape future intentions to mentor, mechanisms that help explain why programs emphasizing reciprocity and support can become self-reinforcing talent pipelines (Kakyo et al., 2025)

### 4.8 Recommendations for program improvement

To enhance the effectiveness of the AGEAP USA Mentoring Program and address the challenges identified, several improvements are recommended. These include refining the mentor-mentee matching process to better align interests and goals, setting clear expectations from the outset to foster accountability, and developing practical resources such as templates, webinars, and how-to guides. Mentees should be given the opportunity to select mentors based on shared career paths or academic interests, while the program should implement greater structural consistency and follow-up mechanisms. Enhancing communication through visual tools like videos and live sessions, creating a centralized web platform for participant management, and offering training to clarify roles and responsibilities for both mentors and mentees are also essential. Finally, establishing continuous monitoring and publicly recognizing mentors and volunteers can help sustain engagement and strengthen the program's long-term impact.

## Data Availability

The raw data supporting the conclusions of this article will be made available by the authors, without undue reservation.
