# Supplementary material for: Outcome harvesting: evaluation of a mentoring program supporting Latino agricultural professionals in the United States
Source: Front Sociol. 2026 May 11;11:1726136. doi: 10.3389/fsoc.2026.1726136 (PMC13199001; doi:10.3389/fsoc.2026.1726136)
Supplement: Supplementary file 1 [file Supplementary_Table_1.docx]

# Appendix

**Table 1.**

*Summary of Research Questions, Themes, and Subthemes*

| **Research Question** | **Theme** | **Subthemes** |
| --- | --- | --- |
| RQ1: How do Participants Perceive Benefits from the AGEAP-USA Mentoring Program?​ | Benefits Perceived by Mentees | 1. Professional Development 2. Learning Through Shared Experiences 3. Expanding Professional Connections |
|  | Benefits Perceived by Mentors | 1. Communication Skills 2. Role Modeling 3. Continuous Learning |
|  | Benefits Perceived by Organizers | 1. Organizational Skills Development 2. Connected Growth |
| RQ2: What Challenges did participants encounter during their Involvement in the AGEAP-USA Mentoring Program? | Lack of Commitment by Mentees | N/A |
|  | Difficulties Establishing Connections | N/A |
|  | Difficulty Recruiting Mentors & Maintaining Commitment​ | N/A |
| RQ3: What motivated Zamorano Alumni to Participate in the AGEAP-USA Mentoring Program from 2020 to 2024? | Mentee Motivations | 1. Seek Professional Guidance 2. Learn from Real Experiences; 3. Overcome Fears and Uncertainties |
|  | Mentor and Organizer Motivations | 1. Contribute to the Zamorano Community 2. Improve Transition Experience 3. Strengthen Sense of Belonging with Zamorano |
| RQ4: To what extent did the program achieve its objectives? | Achievement of Program Objectives | N/A |
